# Urinary Vacuolar Casts Are a Unique Type of Casts in Advanced Proteinuric Glomerulopathies

**DOI:** 10.34067/KID.0000000000000346

**Published:** 2024-01-19

**Authors:** Sarah Rosenbloom, Akanksh Ramanand, Anabella Stark, Vipin Varghese, Dustin Chalmers, Nathan Au-Yeung, Swetha R. Kanduri, Ivo Lukitsch, Jose Antonio T. Poloni, Elizete Keitel, Ana Paula Franz, Carlos Martínez-Figueroa, Abhirup Sarkar, Maia C. Alix-Arbatin, Agnes B. Fogo, Florian Buchkremer, Jay R. Seltzer, Juan Carlos Q. Velez

**Affiliations:** 1 Department of Nephrology, Ochsner Health, New Orleans, Louisiana; 2 Ochsner Clinical School, The University of Queensland, Brisbane, Queensland, Australia; 3 Controllab, Rio de Janeiro, Brazil; 4 Santa Casa de Misericórdia de Porto Alegre, Center for Nephrology and Kidney Transplantation, Porto Alegre, Rio Grande do Sul, Brazil; 5 Universidade Federal de Ciências da Saúde de Porto Alegre, Porto Alegre, Rio Grande do Sul, Brazil; 6 Laboratório de Análises Clínicas, Hospital de Clínicas, Passo Fundo, Rio Grande do Sul, Brazil; 7 Escuela Nacional de Ciencias Quimicobiológicas, IPN, Mexico City, Mexico; 8 Suraksha Diagnostics, Kolkata, India; 9 Cebu Doctors University Hospital, Cebu City, Phillippines; 10 Department of Pathology, Microbiology and Immunology, Vanderbilt University School of Medicine, Nashville, Tennessee; 11 Division of Nephrology, Medical University Department, Kantonsspital Aarau, Aargau, Switzerland; 12 Department of Nephrology, Missouri Baptist Medical Center, St. Louis, Missouri

**Keywords:** urinary sediment, urine microscopy, urine sediment, nephrotic, diabetic, thrombotic microangiopathy, vacuolization, vacuolated, tubular, glomerular, albuminuria, clinical nephrology, acute renal failure, glomerulopathy, glomerulosclerosis, proteinuria, renal pathology

## Abstract

**Key Points:**

- Vacuolar casts are a distinct type of casts identifiable by urinary sediment microscopy.
- Identification of urinary vacuolar casts is associated with the presence of an advanced and severe form of a proteinuric glomerular disease.

**Background:**

Identification of casts by urinary sediment microscopy is a valuable diagnostic clinical tool for the evaluation of kidney disease. Vacuolar casts are an unrecognized unique type of casts characterized by the presence of nonpolarizable, clear vesicles of various sizes contained within a cast matrix, different from lipid casts, erythrocyte casts, or any other casts. We aimed to gain better understanding of the clinical relevance of these casts by establishing a multinational collaborative group to search for cases in which vacuolar casts were identified.

**Methods:**

Leveraging an educational social media platform, we conducted a multinational observational study extracting cases of patients who presented with urinary vacuolar casts during evaluation for impaired kidney function. Parameters assessed included degree of proteinuria and kidney dysfunction, clinical and histopathological diagnosis, and severity of renal parenchymal scarring on biopsy. A control group of patients without vacuolar casts was included for comparison.

**Results:**

Forty-six patients with urinary vacuolar casts were compiled from six countries. Nephrotic range proteinuria (82%), glomerular etiology (98%), and advanced CKD stage (62% 3B-5) were salient features. Histopathological diagnosis was available in 26 (57%) patients. Combining clinical and pathological diagnoses, diabetic nephropathy (48%), arterionephrosclerosis (30%), podocytopathies (15%), and proliferative glomerulonephritides (15%) accounted for most patients. Vacuolization of tubules or podocytes was present in 61% of the specimens. When compared with patients with histopathological diagnoses in which vacuolar casts were not found (*n*=186), patients with vacuolar casts more frequently had a glomerular etiology (100% versus 71%, *P* = 0.002), had greater proteinuria (median urine protein-to-creatinine 10.3 versus 2.2 g/g, *P* < 0.001), and had greater proportion of patients with ≥30% glomerular obsolescence (46% versus 20%, *P* = 0.003).

**Conclusions:**

Thus, urinary vacuolar casts are strongly associated with advanced glomerulopathies with severe proteinuria. Future studies should examine their origin, composition, and prognostic value.

**Podcast:**

This article contains a podcast at https://dts.podtrac.com/redirect.mp3/www.asn-online.org/media/podcast/K360/2024_01_26_KID0000000000000346.mp3

## Visual Abstract

**Figure:**
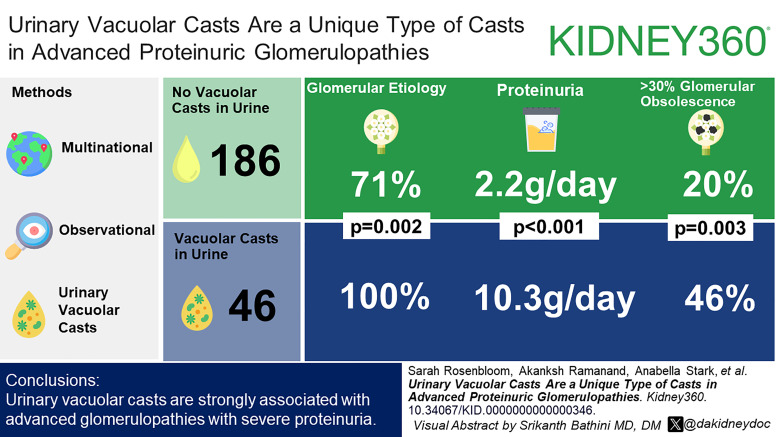

## Introduction

Identification of urinary casts provides clinically useful information for the diagnosis and prognosis of acute and chronic kidney pathologies.^1–6^ In certain clinical contexts, recognition of specific casts can establish a diagnosis or determine the need for a kidney biopsy. Hyaline casts are reflective of tubular stasis, whereas renal tubular epithelial cell (RTEC) casts (RTECC), granular casts, and waxy casts are indicative of a tubular insult.^1,7^ Erythrocyte or red blood cell (RBC) casts and white blood cell casts suggest presence of glomerulonephritis or tubulointerstitial nephritis.^6^ Lipid casts are often identified in the context of nephrotic range proteinuria. Crystals contained in casts are seen in patients of drug-induced tubular damage or formation of crystals (*e*.*g*., calcium oxalate, leucine) in the context of sluggish tubular flow.

In addition to the aforementioned urinary casts, there is a distinct type of urinary cast with a unique morphology characterized by vacuolar inclusions that thus far has not been well reported or studied and is not described in textbooks of urinary sediment microscopy.^8,9^ These casts contain nonpolarizable oval, fluid-filled, lipid-like vesicles immersed within a lightly granular cast matrix. These casts have been termed denatured vacuolar (or vacuolated) acellular casts.^10^ A single case report in the PubMed-indexed medical literature described a patient with advanced CKD and provided representative images of these casts.^10^ It has been hypothesized that vacuolar casts may originate from vacuolated RTECs or modified lipid casts.^10,11^ However, the clinical significance of vacuolar casts remains unclear. In this study, we present the first case series of patients who presented for clinical evaluation of kidney disease and whose urinary specimens contained vacuolar casts.

## Methods

### Study Design

**Figure 1:**
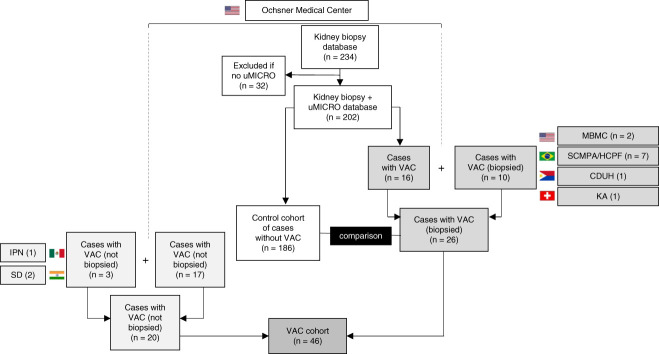
**Study design.** Establishment of a collaborative multinational study to identify patients of vacuolar casts (VAC). Most patients were captured at Ochsner Medical Center. Among 202 patients with kidney biopsy-proven diagnosis and concomitant documentation of urinary sediment microscopy (uMICRO) findings, we identified 16 patients presenting with VAC as well as 186 controls without VAC. Additional ten cases were collected from Missouri Baptist Medical Center (MBMC), Santa Casa de Misericórdia de Porto Alegre (SCMPA), Hospital de Clinicas de Passo Fundo (HCPF), Kantosspital Aarau (KA), and Cebu Doctors University Hospital (CDUH). In addition, 17 patients presented with VAC by uMICRO but did not undergo kidney biopsy. Additional three patients of VAC without biopsy were collected from Instituto Politécnico Nacional (IPN) and Suraksha Diagnostics (SD). Altogether, the VAC cohort comprised 46 patients.

This study was conducted with approval by the Institutional Board Review at each participating institution and in accordance with the Declaration of Helsinki. After encountering several cases of patients with urinary specimens containing vacuolar casts during routine clinical practice in the hospital and clinic settings at Ochsner Medical Center (OMC), we aimed to compile cases to describe their associated clinical and histopathological features. To expand our investigation, we used the open access social media platform X (formerly Twitter) to contact specialists in nephrology and clinical laboratory medicine who frequently post images of urinary sediment microscopy, asking them to provide clinical data and microphotographs of encountered cases of vacuolar casts (Figure 1, also see Supplemental Methods). Microscopic examination of the urinary sediment was performed approximately within 3 hours of specimen collection and as previously described with and without Sternheimer-Malbin (SM) stain (Supplemental Methods).^12^ Age, race, sex, body mass index, diagnosis of diabetes or hypertension, other comorbidities, serum creatinine and other blood chemistry, presence of CKD on the basis of eGFR, presence of AKI, urinalysis, degree of proteinuria, concomitant findings in the urinary sediment, and need for dialysis were collected.

### Vacuolar Casts

**Figure 2:**
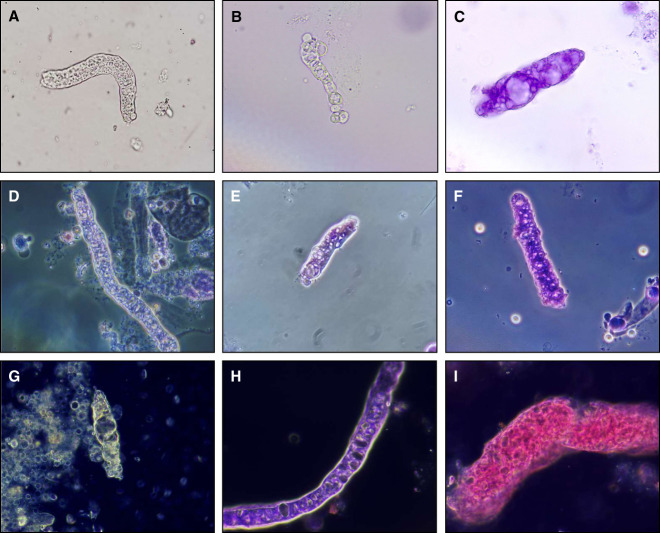
**Representative microphotographs of vacuolar casts.** Casts visualized by bright field illumination (A–C), phase contrast microscopy (D–F), and dark field illumination (G–I). For (C, E, F, H, and I), urine sediment specimens were treated with Sternheimer-Malbin stain. All images captured at 400× magnification.

**Figure 3:**
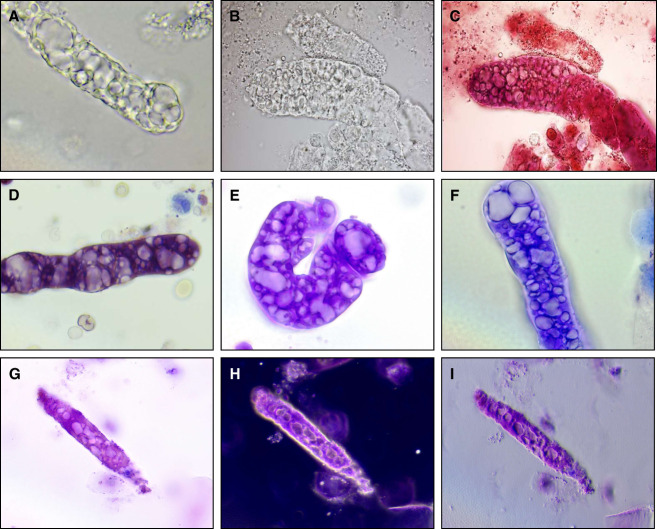
**Representative microphotographs of vacuolar casts at higher magnification.** Casts visualized by bright field illumination (A–G), dark field illumination (H), and oblique illumination (I). (B and C) depict the same cast before and after application of Sternheimer-Malbin (SM) stain. (G–I) depict the same cast visualized by three different modalities (bright, dark, oblique illumination). For (D–I), urine sediment specimens were also treated with SM stain. Images captured at 1000× (A–F) and 500× (G–I) magnification. Oblique illumination images created the impression that the vacuoles represent depressions or concavities within the casts rather fluid-filled vesicles. However, the three-dimensional views generated by this technique may merely be artifactual.

**Figure 4:**
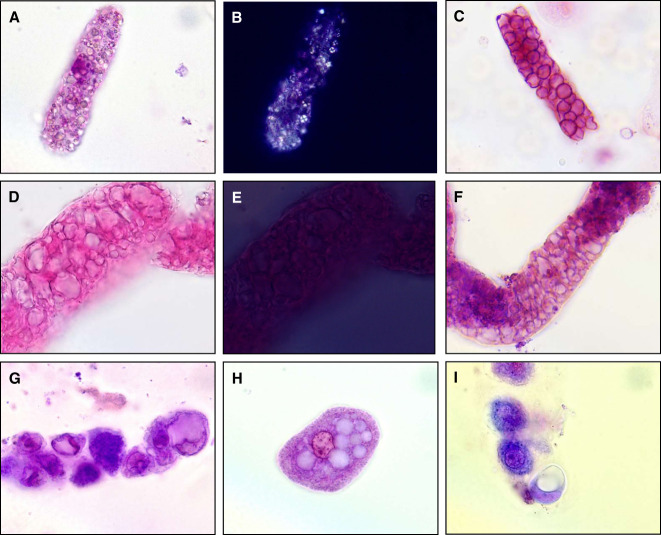
**Comparison of vacuolated cast with lipid casts and red blood cell (RBC) casts.** Images in middle row (D–F) are all of vacuolar casts. Photomicrograph of a lipid cast imaged at bright field illumination (A) and under polarized light (B), the latter depicting the Maltese cross sign. Vacuolar cast also imaged at bright field illumination (D) and under polarized light (E) is shown right below the lipid cast for comparison. Note that the vesicles within the vacuolar cast do not produce a Maltese cross sign. Photomicrograph of an RBC cast is shown (C) above another vacuolar cast (F), both imaged at bright field illumination. Note that RBC are round and of similar size, whereas vacuolar inside vacuolar casts adopt various shapes and sizes. In proteinuric states, renal tubular epithelial cells (RTEC) often contain large vacuoles. Representative examples of an RTEC cast with vacuolated RTECs (G) and free-floating vacuolated RTECs (H–I) at bright field illumination. All urine sediment specimens treated with Sternheimer-Malbin stain. All images captured at 1000× magnification.

A case of vacuolar casts valid for inclusion in this case series was considered when a photomicrograph demonstrated the following characteristics: (*1*) recognizable cast matrix; (*2*) presence of multiple nonpolarizable, membrane-delimited, fluid-filled, clear vesicles of variable size within the cast matrix and outside of cells; (*3*) verification that the cast identified was not a lipid (fatty) cast using polarized light, or a RBC cast on the basis of morphology (Figures 2–4). It should be noted that some vacuolar casts contained isolated cells or lipid droplets within the matrix of the cast in addition to the free-standing vacuoles. Validity was further verified by at least two other coauthors of the article who reviewed the microphotographs.

### Histopathology

Tissue specimens were evaluated as per routine clinical practice under light microscopy, immunofluorescence, and electron microscopy (EM). Specimens were assessed for degree of interstitial fibrosis and tubular atrophy (IFTA) and glomerular obsolescence and for presence of foam cells or vacuolization in tubules or podocytes.

### Control Cohort

To assess the significance of the histopathological findings associated with patients presenting with urinary vacuolar casts, we established a control cohort that consisted of patients at OMC who underwent kidney biopsy during the same study period, had documented urine microscopy at the time of biopsy, and lacked vacuolar casts. To address a confounder by diagnosis, we performed a secondary comparison by selecting a subgroup restricted to patients with a pathological diagnosis that matched a pathological diagnosis identified in the vacuolar casts' cohort.

## Results

### Identification of Patients with Vacuolar Casts

We identified vacuolar casts in 46 individual patients from a total of eight institutions from four continents (Figure 1). In most instances, vacuolar casts were not abundantly present on each specimen: 1–2 vacuolar casts were found in 5%– 20% of low power fields. The vacuoles within casts were of variable shape and size, most of them relatively round and with a diameter range of approximately 2–25 *µ*m.

### Patient Characteristics

Median age was 64 (15–82) years, and 41% were women. Race included White (37%), Black (28%), Hispanic (22%), and Asian (13%) patients. Thirty-three (85%) patients had known CKD at baseline, and 24 (62%) had either stage 3B, 4, or 5 CKD, whereas the baseline kidney function was unknown in seven patients. Six (15%) patients presented with *de novo* AKI without preexisting CKD, 13 (33%) presented with AKI superimposed on CKD, and 20 (51%) presented as progressive CKD. The median baseline serum creatinine was 1.5 mg/dl (0.8–3.3). At the time of identification of vacuolar casts, the median serum creatinine was 3.1 mg/dl (1.2–7.4). Regarding comorbidities, 32 (70%) had hypertension, and 23 (50%) had type 2 diabetes mellitus (Table 1). Of those with reported hemoglobin A1c levels (*n*=31), an A1c >8.0% were found in eight patients (26%). After 6 months of follow-up, 13 (28%) patients reached ESKD necessitating dialysis.

**Table 1:** Demographic and clinical characteristics and blood laboratory values of patients with vacuolar casts (*n*=46)

Parameter	*n* (%)
Age (yr)	64 (15–82)
**Race, *n* (%)**	
White	17 (37)
Black	13 (28)
Asian	6 (13)
Hispanic	10 (22)
**Sex, *n* (%)**	
Male	27 (59)
Female	19 (41)
Body mass index (kg/m^2^)^a^	26 (19–47)
Baseline sCr (mg/dl)^a^	1.5 (0.8–3.3)
**Baseline CKD stage**	33 (85)
3A	11 (24)
3B	8 (17)
4	13 (28)
5	8 (17)
Unknown	7 (15)
sCr at presentation (mg/dl)	3.1 (1.2–7.4)
*De novo* AKI	6 (15)
AKI on CKD	13 (33)
Progressive CKD	20 (51)
**Comorbidities**	
Type 2 diabetes mellitus	23 (50)
Hypertension	32 (70)
Albumin (g/dl)^a^	2.3 (1.7–3.9)
Total cholesterol (mg/dl)^a^	179 (45–296)
LDL cholesterol (mg/dl)^a^	95 (39–196)

Data presented as *n* (%) or median (range). sCr, serum creatinine.

a *n*=39.

### Urine Laboratory Tests and Concomitant Findings in the Urinary Sediment

Dipstick proteinuria was positive in all but one patient (suspected patient of myeloma cast nephropathy), *i*.*e*., in 98%; 2+ proteinuria in 10 (22%), and 3+ proteinuria in 35 (76%). Among 38 (84% of the cohort) patients who had urine protein-to-creatinine ratio (UPCR) available, 31 (82%) had nephrotic range proteinuria. The median UPCR was 6.1 g/g (1.2–28.2). Including vacuolar casts, at least three different types of casts were found in 39 (85%) patients (Table 2). Overall, vacuolar casts were identified among other casts in all (100%) patients.

**Table 2:** Urine laboratory data of patients presenting with vacuolar casts (*n*=46)

Parameter	*n* (%)
**Dipstick urine protein**	
Negative	1 (2)
1+	0 (0)
2+	10 (22)
3+	35 (76)
**RBC (cells/hpf)**	
0–2	14 (30)
3–20	18 (39)
21–99	7 (15)
>100	7 (15)
**WBC (cells/hpf)**	
0–2	14 (31)
3–20	25 (54)
21–49	2 (4)
>50	5 (11)
**UPCR (g/g)** ^a^	6.1 (1.5–28.2)
1–3.4	7 (18)
3.5–10	20 (51)
>10	12 (31)
**Years of proteinuria**	
Unknown	19 (41)
0–5	15 (33)
6–10	4 (9)
>10	8 (17)
**Concomitant urinary sediment microscopy findings**	
Casts	45 (100)
*Hyaline*	16 (35)
*Granular*	31 (67)
*Waxy*	30 (65)
*RTEC*	18 (39)
*WBC*	16 (35)
*RBC*	11 (24)
Lipids (oval fat bodies and lipid casts)	19 (41)
Cells	45 (100)
*WBC*	31 (67)
*RBC*	32 (70)
*Acanthocytes*	13 (28)
*RTEC*	18 (39)

Data presented as *n* (%) or median (range). RBC, red blood cells; RTEC, renal tubular epithelial cell; UPCR, urine protein-to-creatinine; WBC, white blood cells.

a *n*=39.

### Histopathology

**Figure 5:**
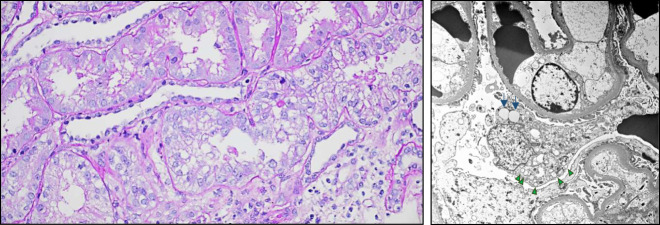
**Representative photomicrographs illustrating vacuolization of tubuli and podocytes identified in tissue specimens from patients who presented with urinary vacuolar casts.** (A) Numerous vacuoles are present in proximal tubules with partial loss of brush borders (light microscopy, periodic acid–Schiff stain, original magnification 40×). (B) Podocyte with multiple vacuoles (clear spaces, green arrowheads) and occasional lipid inclusions (light gray, left, blue arrows) (transmission electron microscopy, original magnification 3500×).

Twenty-six of the 46 (57%) patients underwent kidney biopsy (Table 3). Within this group, all (100%) patients were found to have a glomerular lesion. In 14 (54%) patients, there was evidence of two glomerular diseases in the same tissue specimen. Greater than 30% IFTA was present in 14 (56%) patients. Regarding glomerular obsolescence, the median percentage of globally sclerotic glomeruli was 30% (5–75), with 12 (46%) patients exhibiting >30%. Vacuolization and apical blebbing of tubular epithelia and/or tubular intracytoplasmic protein droplets were found in 8 (31%) patients (Figure 5). Either interstitial or glomerular foam cells were reported in 3 (12%).

**Table 3:** Histopathological findings in patients with vacuolar casts who underwent kidney biopsy (*n*=26)

Histopathological Diagnosis	*n* (%)
Diabetic nephropathy	7 (27)
Arterionephrosclerosis	6 (23)
Thrombotic microangiopathy	4 (15)
**Podocytopathy**	7 (27)
Focal segmental glomerulosclerosis	3 (12)
Focal global glomerulosclerosis	1 (4)
Minimal change disease	1 (4)
Collapsing glomerulopathy associated with COVID-19	1 (4)
Membranous nephropathy	1 (4)
IgA nephropathy	3 (12)
**Proliferative glomerulonephritis**	7 (27)
Diffuse proliferative glomerulonephritis	4 (13)
*Hepatitis C–associated (crescentic)*	1 (4)
*Infection-related (crescentic)*	1 (4)
*C3-dominant*	2 (8)
Lupus nephritis ISN/RPS class IV	2 (8)
Proliferative glomerulonephritis with monoclonal IgG kappa deposits	1 (4)
Idiopathic nodular glomerulosclerosis	1 (4)
ANCA-associated pauci-immune glomerulonephritis	1 (4)
Amyloidosis	1 (4)
Fibrillary glomerulonephritis	1 (4)

Percentages add to more than 100% because of patients with two or more diagnoses. COVID-19, coronavirus disease of 2019; ISN, International Society of Nephrology; RPS, Renal Pathology Society.

Tissue specimens were examined by EM in 18 of the 26 (69%) patients. Podocyte foot process effacement was present in all (100%) patients, with a median percentage of 90% (50–100). Podocyte vacuolization or intracytoplasmic protein droplets were found in 7 (39%) patients (Figure 5). Combining light microscopy and EM, 11 (61%) patients had evidence of either vacuolization of tubules or podocytes or presence of interstitial or glomerular foam cells.

### Clinical Diagnoses

Clinical diagnosis of diabetic nephropathy without biopsy confirmation was made in 15 (33%) patients. Similarly, clinical diagnosis of hypertension-associated arterionephrosclerosis was made in 8 patients, thrombotic microangiopathy (TMA) in one, and myeloma cast nephropathy in one (only patient with presumed tubular etiology). Grouping clinical and histopathological data, the most common overall diagnoses were diabetic nephropathy in 22 (48%), arterionephrosclerosis in 14 (30%), podocytopathies in 7 (15%), proliferative glomerulonephritides in 7 (15%), and TMA in 5 (11%). Two overlapping diagnoses were assigned to 18 (39%) patients.

### Control Cohort

Among 202 patients who underwent kidney biopsy and completed urine sediment microscopy at OMC, 16 (8%) had vacuolar casts, and 186 (92%) did not (Figure 1). Nine additional patients from other sites added to a total of 25 patients with vacuolar casts and histopathological diagnosis. Among the 186 control patients, 132 patients (71%) had a glomerular lesion, and 54 (29%) had a nonglomerular diagnosis (acute tubular injury, tubulointerstitial nephritis, pyelonephritis) (Supplemental Table 2). This distribution was markedly different than that of the vacuolar cast cohort which consisted of 100% glomerular diseases (71% versus 100%, *P* = 0.002). Ninety patients (47%) in the control group had preexisting CKD, compared with 17 (65%) in the vacuolar cast cohort (*P* = 0.09) (Supplemental Table 1).

**Figure 6:**
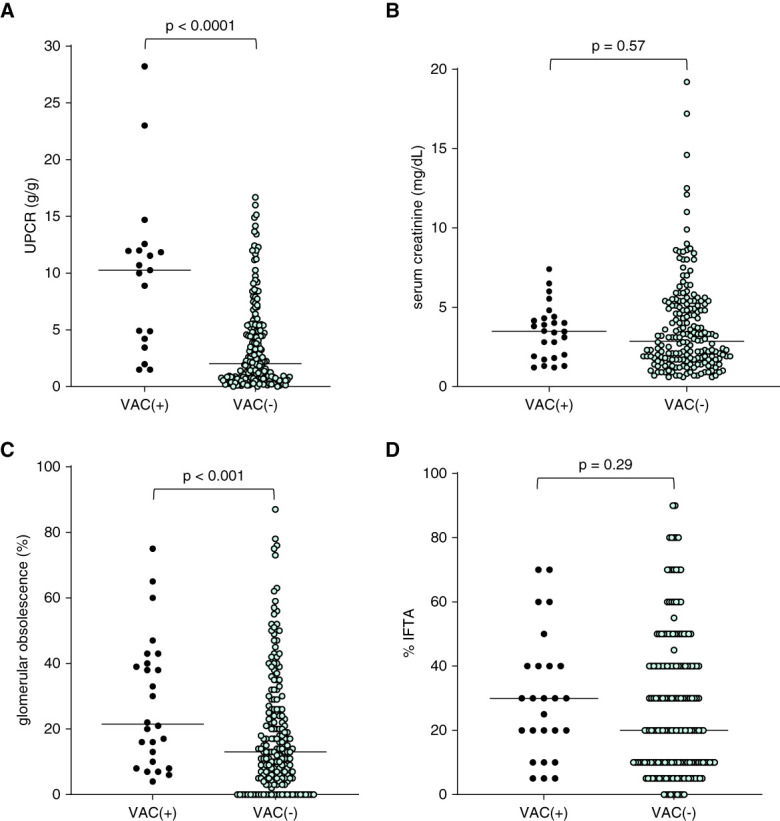
**Degree of proteinuria, kidney function, and histopathological features of patients with vacuolar casts [VAC(+)] (*n*=25) compared with a control group without VAC [VAC(−)] in the urinary sediment (*n*=186).** (A) Median urine protein-to-creatinine (UPCR) ratio was significantly greater in the VAC(+) group. (B) No significant difference was observed between groups in serum creatinine concentration. (C) The median percentage of glomerular obsolescence was significantly greater in the VAC(+) group. (D) No significant difference was observed between groups in the degree of interstitial fibrosis and tubular atrophy (IFTA).

**Figure 7:**
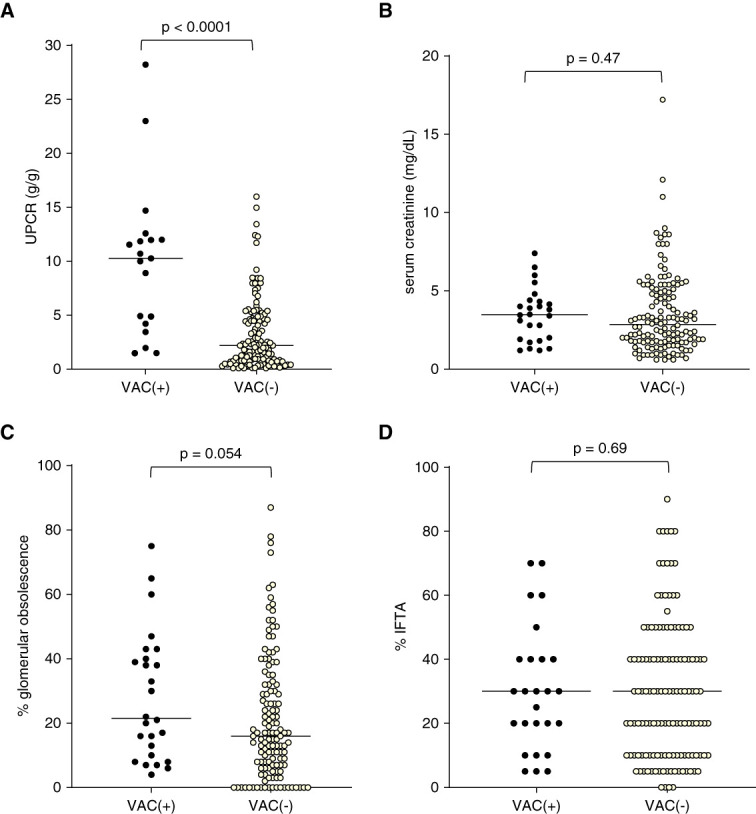
**Degree of proteinuria, kidney function, and histopathological features of patients with vacuolar casts [VAC(+)] (*n*=25) compared with a subgroup of patients with biopsy-proven glomerulopathies without VAC [VAC(−)] in the urinary sediment (*n*=132).** (A) Median UPCR ratio was significantly greater in the VAC(+) group. (B) No significant difference was observed between groups in serum creatinine concentration. (C) The median percentage of glomerular obsolescence was significantly greater in the VAC(+) group. (D) No significant difference was observed between groups in the degree of IFTA.

There was no significant difference in median serum creatinine among groups (3.5, 2.8 and 2.8 mg/dl, for the vacuolar cast group, control group [*P* = 0.57], and subgroup control group restricted to glomerulopathies [*P* = 0.47], respectively) (Figures 6 and 7). The median UPCR in the vacuolar cast group (10.3 g/g) was significantly greater than that of the control (2.2 g/g, *P* < 0.0001) and the subgroup control (2.6 g/g, *P* < 0.0001) cohorts (Figures 6 and 7). There was no significant difference in the proportion of patients with ≥30% IFTA between groups (52%, 46% and 49%) or in median percentage of IFTA (30%, 20%, and 30%) for the vacuolar cast, control, and subgroup control cohorts, respectively. On the other hand, the median percentage of glomerular obsolescence was greater among those with vacuolar casts (22%) compared with the control (13%, *P* < 0.0001) and subgroup control (16%, *P* < 0.0001) cohorts lacking vacuolar casts (Figures 6 and 7). Furthermore, the proportion of patients with ≥30% glomerular obsolescence was greater for those with vacuolar casts (46%) compared with the control (20%, *P* = 0.0032) or subgroup control (27%, *P* = 0.054) cohorts, whereas zero patients with vacuolar casts had 0% glomerular obsolescence compared with 16% of control (*P* = 0.028) and 15% of the subgroup control (*P* = 0.035) groups lacking vacuolar casts (Supplemental Figure 1).

### Sudan III Staining

**Figure 8:**
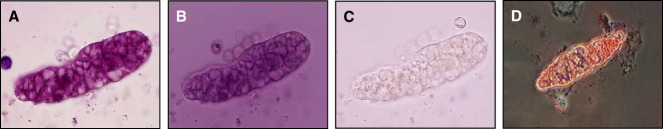
**Sudan III staining of vacuolar casts**. (A–C) Snapshots of appearance of a vacuolar cast at bright field illumination (1000×) in a specimen treated with SM stain (A). On application of Sudan III stain, displacement of SM stain is rapidly observed at 5 seconds (B) and 20 seconds (C). No immediate uptake of Sudan III stain within the vacuoles is seen. (D) Microphotograph of a vacuolar cast from a different case visualized by phase contrast microscopy (800×). Uptake of Sudan III in the cast matrix around the vacuoles is seen after 20 minutes. Negligible uptake of Sudan II stain is observed within the vacuoles.

No clear uptake of the Sudan III stain for lipids was observed inside the vacuoles (Supplemental Methods). In one instance, we observed uptake of Sudan III stain in a cast matrix containing vacuoles. However, minimal to no uptake of stain was observed within vacuoles. In addition, on applying Sudan III stain to the edge of the coverslip of an SM-stained slide, it was observed that Sudan III stain caused the SM stain to evacuate the observed cells and casts without evidence of uptake of stain by the vacuoles. When the SM stain left the cast, it was noted that the vacuoles seemed to display cytoplasmic granular movement (Figure 8).

## Discussion

This investigation constitutes the first of its kind reporting a cohort of patients who, on clinical evaluation, were found to have urinary sediment specimens containing a unique type of casts named vacuolar casts. The overwhelming majority of patients in which these casts were identified had evidence of having advanced CKD of glomerular etiology or severe AKI also of glomerular etiology. Virtually all the patients presented with overt proteinuria, with over 80% of them in the nephrotic range. Among those who underwent kidney biopsy, a glomerulopathy was always present, and in most cases, a moderate-to-severe degree of IFTA and globally sclerotic glomeruli were observed. The most common diagnoses were diabetic nephropathy, arterionephrosclerosis, podocytopathy, proliferative glomerulonephritis, and TMA. A large proportion of patients in the cohort either presented at advanced stages of CKD or progressed toward ESKD. Therefore, vacuolar casts are a type of urinary cast associated with severe, advanced, and progressive proteinuric glomerular disease.

Although granular casts can be found in the context of CKD and proteinuria, they are more commonly found in reversible cases of AKI because of acute tubular injury without glomerular involvement.^1,3,7,12,13^ Waxy casts have been associated with CKD.^14–16^ However, waxy casts can be identified in nonglomerular forms of CKD, in patients of acute tubular injury, and in the absence of overt proteinuria.^7,15,17^ Thus, vacuolar casts seem to be more specific indicators of presence of either a chronic or a severe acute proteinuric glomerular disease. In addition, in most patients, vacuolar casts are found in specimens that also contain other acellular and cellular casts, highlighting the complexity of the conditions in which vacuolar casts emerge. Therefore, identification of vacuolar casts may be of value for diagnostic approaches. For instance, identifying vacuolar casts might help clinicians weigh on decisions regarding pursuance of a kidney biopsy in patients where chronicity and suitability for immunosuppression is in question.

Before this report, only one publication describing vacuolar casts was found in PubMed. Martinez-Figueroa *et al.* described a 62-year-old man with type 2 diabetes mellitus and CKD stage 5 on peritoneal dialysis.^4^ A urine specimen was submitted to the laboratory under suspicion of a urinary tract infection. The specimen revealed 3+ dipstick proteinuria and hematuria. In addition, it contained oval fat bodies and a large variety of other casts.^4^ Histopathology was not available. In agreement with our findings, a report of the Japanese Association of Medical Technologists (not in PubMed) described vacuolar casts as occurring in patients of diabetic nephropathy with severe proteinuria or reduced kidney function.^11^ Thus, our report is aligned with previous isolated reports and expands on the clinical phenotype and pathological characteristics associated with vacuolar casts.

Lipid casts or oval fat bodies are known indicators of nephrosis.^18^ However, they can be detected in any podocytopathy presenting with nephrotic-range proteinuria regardless of severity or chronicity. Lipid casts and oval fat bodies can be identified in a patient presenting with normal kidney function and with fully reversible nephrotic syndrome. By contrast, vacuolar casts seem to form in more aggressive cases. None of the patients in this cohort had normal kidney function or reversible nephrotic syndrome. The common denominator was presence of overt proteinuria in the context of either severe AKI or advanced CKD. Our single case of myeloma cast nephropathy presenting with vacuolar casts is intriguing. Perhaps cell perturbation by light chain globulinuria may lead to the formation of these casts.

When comparing the degree of proteinuria observed in patients without vacuolar casts, those with vacuolar casts had significantly greater proteinuria and a greater degree of glomerular obsolescence (Figure 7). Both severe proteinuria and glomerular obsolescence are associated with an increased risk for progression to ESKD.^19,20^ A significant proportion of patients in our cohort progressed to ESKD. Therefore, vacuolar casts may be considered an indicator of presence of a severe proteinuric glomerular disease with high risk for progression to ESKD.

Because of the association of vacuolar casts with severe and advanced glomerulopathies, it is important to correctly identify them during performance of microscopic examination of the urinary sediment for the evaluation of a case of AKI or CKD. Lipid casts typically contain multiple round lipid droplets of various sizes, and the largest droplets often produce a Maltese cross sign when inspected under polarized light (Figure 4). Unlike lipid casts, vacuolar casts contain vesicles of various shapes; some may be round, but other vacuoles can adopt oval or elongated shapes. Furthermore, vacuolar casts do not polarize, unless they also contain lipid droplets immersed among the vacuoles. RBC casts are known indicators of nephritic conditions, such as proliferative and pauci-immune glomerulonephritides. Therefore, they convey clinical information that is vastly different than that conveyed by vacuolar casts. RBC casts typically contain multiple contiguous RBCs of a round shape. Although RBCs can occasionally adopt an oval shape when they are confined to a restricted space within casts, the size of each RBC is usually uniform. On the other hand, vacuoles within vacuolar casts can be significantly smaller or larger than the size of RBCs (Figure 4). Despite these differences, misinterpretation is possible. Thus, it is encouraged to use all available microscopy techniques (bright field, dark field, phase contrast, polarized light, 100–1000×, SM stain) available to correctly identify vacuolar casts. Bright field illumination provides the highest resolution. Dark field illumination is helpful for a quick scanning of the slide at low magnification. Phase contrast microscopy allows for visible outlining of the vacuoles. SM stain aids in visualizing the cast matrix and distinguishing the vacuoles from other structures. Polarized light generates a characteristic signal for lipids.

The origin of vacuolar casts remains unknown. Our data suggest that the vacuoles likely do not contain LDL cholesterol, which typically polarizes.^21^ It has previously been hypothesized that vacuolar casts occur when the lipid component of cells or fatty casts is lost.^10,11^ One possibility is that vacuolar casts originate from degenerated vacuolated RTECs trapped inside casts. Several elements are in support of this hypothesis: (*1*) Concomitant presence of RTECs and RTECCs was found in the urinary specimens containing vacuolar casts; (*2*) nonpolarizable vacuolated RTECs (inside or outside casts) are often found in patients with nephrotic range proteinuria presumably denoting associated tubular cellular stress/injury or engulfment of filtered protein and/or lipids; (*3*) nephrotic range proteinuria was a predominant feature in our series; (*4*) tubular vacuolization was seen in several of our patients (Figure 5). Thus, vacuolar casts may originate from vacuolated RTECCs in which their cellular membranes break down, the cell degenerates, and vacuoles form and coalesce. Vacuolated RTECs have been reported in patients evaluated for parenchymal kidney disease and have been named bubble cells.^22^ The vesicles in bubbles cells were described as aqueous rather than lipidic and likely resulting from dilated endoplasmic reticuli.^22^

Potentially, a similar phenomenon can occur with other cell types, including RBCs and white blood cells, or foam cells. Foam cells are a cell type of macrophage lineage characterized by cytoplasmic vesicles that are often seen in several podocytopathies or proteinuric glomerulopathies.^23,24^ Interstitial or intraglomerular foam cells were reported in the kidney biopsies in a few patients in our cohort. Another consideration is whether vacuolar casts could originate from podocyturia. Podocyte injury is manifested histologically as foot process effacement and microvillous transformation, but also with appearance of intracytoplasmic electron lucent transport vesicles or formation of pseudocysts as a result of detachment from the glomerular basement membrane.^25,26^ Podocyte vacuolization was present in several patients in our series (Figure 5). Thus, it could be speculated that remains of filtered vacuolated injured podocytes may disintegrate and get trapped within casts. On the basis of our findings using Sudan III stain, it is plausible that the vacuoles consist of primarily aqueous material, but we cannot discard the possibility of them containing modified lipids. It remains unclear whether there are different species of vacuolar casts, wherein some contain lipidic matrices and others may contain aqueous cytoplasmic materials or if their composition changes throughout the lifespan of the cast.

Our report has limitations. Because the cohort was not built prospectively, we were unable to precisely estimate how frequently these casts can be identified in patients with proteinuric CKD. As the authors at OMC (microscopists) became aware of the existence of these casts, more time was occasionally spent examining slides of patients with overt proteinuria toward the later part of the study period, potentially introducing a bias. However, at least 36 low power fields per slide were inspected in all patients (see Supplemental Methods). Histopathological diagnosis, baseline CKD status, and UPCR were not available in all patients. However, the consistent clinical features observed in the entire cohort strongly suggest that the recognized clinical phenotype accurately represents patients with vacuolar casts. Because vacuolar casts are seldom abundant, we cannot rule out the possibility that some of the specimens of the control group may have contained vacuolar casts that were not captured. However, that could also strengthen our observations. The small number of patients per each diagnosis precludes asserting whether any glomerular lesion might be more likely to manifest with vacuolar casts. Nevertheless, the observed enrichment of patients of TMA in our series deserves further exploration. Strengths of this series include the novelty of the observation, the multispecialty, multinational and multiethnic source of our data collection, and its relevant clinical association.

Vacuolar casts constitute a unique type of urinary casts that must be added to a list of urinary casts relevant to human medicine. They can be identified specifically in the context of overt proteinuria, particularly in the nephrotic range, in patients presenting with significant kidney dysfunction, either advanced CKD or severe AKI, due to an aggressive form of a glomerular disease. We suggest their presence may be an ominous prognostic marker. Future studies are needed to determine the origin and composition of these casts, as well as to further explore their prognostic value and clinical utility in the evaluation of individuals with proteinuric AKI or CKD of glomerular etiology.

## Supplementary Material

SUPPLEMENTARY MATERIAL

## Data Availability

All data are included in the manuscript and/or supporting information.

## Supplemental Material

Supplemental Methods.

Supplemental Table 1. Demographic and clinical characteristics and blood laboratory values of patients who underwent kidney biopsy.

Supplemental Table 2. Histopathological findings in the control group of patients without vacuolar casts in the urinary sediment (*n*=186).

Supplemental Figure 1. Degree of glomerulosclerosis.
