# Supplementary material for: Urinary Vacuolar Casts Are a Unique Type of Casts in Advanced Proteinuric Glomerulopathies
Source: Kidney360. 2024 Jan 19;5(2):216–27. doi: 10.34067/KID.0000000000000346 (PMC10914204; doi:10.34067/KID.0000000000000346)
Supplement: SUPPLEMENTARY MATERIAL [file kidney360-5-216-s001.pdf]

SUPPLEMENTARY METHODS

Case compilation

Cases (from 2019 – 2023) were collected from Ochsner Medical Center (OMC), Louisiana, USA; Missouri Baptist Medical Center (MBMC), Missouri, USA; Santa Casa de Misericórdia de Porto Alegre (SCMPA), Porto Alegre, Brazil; Hospital de Clinicas de Passo Fundo (HCPF), Passo Fundo, Brazil; Suraksha Diagnostics (SD), Kolkata, India; Cebu Doctors University Hospital (CDUH), Cebu City, Philippines; Kantonsspital Aarau (KA), Switzerland; and Instituto Politécnico Nacional (IPN), Mexico City, Mexico.

Urine Microscopy

At the primary site at OMC as well as at MBMC, CDUH and KA, specimens were collected from patients evaluated by the nephrology consultation team. At IPN, SD, SCMPA and HCPF, specimens were inspected in a general hospital or stand-alone laboratory. Once collected, specimens were kept at room temperature and transferred to the laboratory for processing. A 10 ml aliquot of urine was placed in a 15 ml high-clarity polypropylene conical tube and centrifuged at 600 g for 5 mins. The supernatant was poured off and the pellet was resuspended by manual agitation in the remaining 0.2 ml of supernatant. A plastic transfer pipette was used to place a single drop onto a standard microscope slide and a coverslip was placed over it. This process was done with and without Sternheimer-Malbin stain[1] (Kova®, Garden Grove, CA, USA). Then, each sample was examined by a trained operator with 10x, 40x, 50x and 100x magnification objectives and a 10x magnification eyepiece, using bright field, dark field and oblique illumination, and phase contrast microscopy. A minimum of 36 low power fields per slide were inspected. Polarized light was applied in all cases. At OMC, photomicrographs were acquired using a Laxco™ LMC4-BF166 microscope (Mill Creek, WA, USA), a Laxco™ SeBaCam digital camera and Laxco™ SeBaView software and stored in a protected virtual server.

Sudan III staining

Sudan III stain was used to identify lipids in casts[2]. In a subset of cases, 5 - 7 drops of Sudan III stain were added to an aliquot of the urine sediment pellet. The stained sample rested for 15 - 20 minutes to allow the casts to absorb the stain before the sample was plated and inspected as detailed above.

References

1. Sternheimer R, Malbin B. Clinical recognition of pyelonephritis, with a new stain for urinary sediments. *Am J Med*. Sep 1951;11(3):312-23. doi:10.1016/0002-9343(51)90168-4
2. Parrish AE, Alpert LK. Stain for lipid bodies in urinary sediment. *J Am Med Assoc*. Aug 29 1953;152(18):1713. doi:10.1001/jama.1953.63690180003009a

**Table S1.** Demographic and clinical characteristics, and blood laboratory values of patients who underwent kidney biopsy			
	Study Cohort with Vacuolar Casts (n = 26)	Control Cohort without Vacuolar Casts (n = 186)	Control Cohort without Vacuolar Casts Excluding Non-Glomerular Etiologies (n = 132)
Age (years)	64 (15 - 82)	58 (18 – 81)	60 (24 – 81)
Race, n (%)			
White	9 (35)	84 (44)	57 (43)
Black	5 (19)	76 (40)	53 (40)
Asian	3 (12)	10 (5)	8 (6)
Hispanic	9 (35)	19 (10)	12 (9)
Native American	0 (0)	2 (1)	2 (2)
Sex, n (%)			
Male	15 (58)	94 (49)	67 (51)
Female	11 (42)	97 (51)	65 (49)
Baseline sCr (mg/dL) ^	1.3 (0.7 – 3.3)	1.3 (0.6 – 3.6)	1.3 (0.7 – 318)
Baseline CKD stage	17 (65)	90 (47)	65 (49)
Unknown	7 (27)	2 (1)	2 (2)
2	0 (0)	3 (2)	1 (1)
3A	7 (27)	34 (18)	26 (20)
3B	5 (29)	28 (15)	21 (16)
4	3 (12)	25 (13)	17 (13)
5	3 (12)	0 (0)	0 (0)
sCr at presentation (mg/dL)	3.5 (1.2 – 7.4)	2.8 (0.7 – 19.2)	2.8 (0.8 – 17.2)
De novo AKI	2 (8)	99 (52)	65 (49)
AKI on CKD	8 (38)	55 (29)	37 (28)
UPCR (g/g) ^	10.3 (1.5 – 28.2)	2.2 (0.1 – 22.1)	2.6 (0.1 – 22.1)
Co-morbidities			
Type 2 diabetes mellitus	8 (31)	76 (40)	57 (43)
Hypertension	16 (62)	145 (76)	103 (78)

Data presented as n (%) or median (range). ^n = 19. sCr: serum creatinine, CKD: chronic kidney disease, AKI: acute kidney injury, UPCR: urine protein-to-creatinine ratio.

**Table S2:** Histopathological findings in the control group of patients without vacuolar casts in the urinary sediment (n = 186)	
**Histopathological diagnosis**	**n (%)**
Glomerular diseases	132 (71%)
Diabetic nephropathy	26 (14)
Arterionephrosclerosis	16 (9)
Thrombotic microangiopathy	5 (3)
Podocytopathy	35 (19)
Focal segmental glomerulosclerosis	4 (2)
Focal global glomerulosclerosis	2 (1)
Minimal change disease	5 (3)
Collapsing glomerulopathy associated with COVID-19	3 (2)
Membranous nephropathy	16 (9)
Lupus nephritis ISN/RPS class V	5 (3)
IgA nephropathy	21 (11)
Proliferative glomerulonephritis	23 (12)
Diffuse proliferative glomerulonephritis	5 (3)
Hepatitis C-associated (crescentic)	1(1)
Infection-related (crescentic)	3 (2)
C3-dominant	1 (1)
Lupus nephritis ISN/RPS class III-IV	18 (10)
ANCA-associated pauci-immune glomerulonephritis	17 (9)
Amyloidosis	2 (1)
Alport syndrome / Thin basement membrane lesion	2 (1)
Non-glomerular lesions	54 (29%)
Tubulointerstitial diseases	23 (12)
Interstitial	15 (8)
Pyelonephritis	4 (2)
Lymphoproliferative infiltration	3 (2)
IgG4-related diseease	1 (1)
Acute tubular injury	18 (10)
Light chain tubulopathy*	4 (2)
Vascular	9 (5)
Arteriosclerosis	5 (3)
Endothelial cell injury^	4 (2)

COVID-19: coronavirus disease of 2019, ANCA: anti-neutrophilic cytoplasmic antibody, ISN: International Society of Nephrology; RPS: Renal Pathology Society. *includes myeloma cast tubulopathy; ^includes calcineurin inhibitor nephrotoxicity.

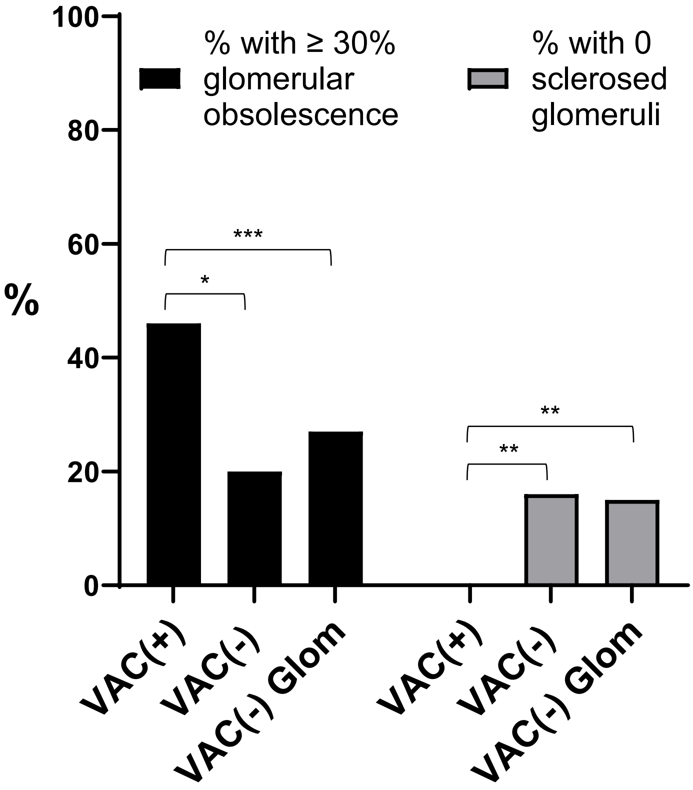

Figure S1 | Degree of glomerulosclerosis. There was a greater proportion of VAC(+) patients with ≥ 30% glomerular obsolescence (46%) compared to the overall VAC(-) control group (20%) as well as the subgroup control group with glomerulopathies and without vacuolar casts [VAC(-) Glom] (27%). No patients in the VAC(+) group lacked globally sclerosed glomeruli (0%), compared to 16% in the VAC(-) group and 15% in the VAC(-) Glom subgroup. *$p < 0.01$; **$p < 0.05$; ***$p = 0.054$.
